# Intralipid postconditioning in patients of cardiac surgery undergoing cardiopulmonary bypass (iCPB): study protocol for a randomized controlled trial

**DOI:** 10.1186/s13063-020-04854-6

**Published:** 2020-11-23

**Authors:** Yuan Yuan, Hui Xiong, Yan Zhang, Hong Yu, Rong-Hua Zhou

**Affiliations:** Department of Anesthesiology, West China Hospital of Sichuan University, No.37, Guoxue Xiang, Wu Hou District, Chengdu, 610041 Sichuan China

**Keywords:** Randomized controlled trials, Intralipid, Postconditioning, Cardiopulmonary bypass, Cardiac surgery, Ischemia/reperfusion injury, Outcome

## Abstract

**Background:**

Intralipid is a necessary fatty acid carrier that has been safely used as an energy supplier in the clinic. It has played an important role in rescuing the cardiac arrest caused by local anesthetic toxicity. In recent years, experimental studies have shown that intralipid postconditioning (ILPC) could reduce myocardial ischemic/reperfusion (I/R) injuries. Our research group has innovatively conducted a pilot randomized controlled trial (RCT), and the results showed that ILPC could reduce the release of cTnT and CK-MB, biomarkers of myocardial I/R injury, in valve replacement surgery. However, the potential effects of ILPC on the clinical outcome of adult cardiac surgery patients are unclear. Intralipid postconditioning in patients of cardiac surgery undergoing cardiopulmonary bypass (iCPB) trial is aimed to further study whether ILPC could improve short-term and long-term clinical outcome, as well as cardiac function in adult cardiac surgery patients.

**Methods:**

The iCPB trial is an ongoing, single-center, prospective, double-blinded, large sample RCT. In total, 1000 adults undergoing cardiac surgery will be randomly allocated to either the ILPC group or the control group. The intervention group received an intravenous infusion of 2 mL/kg of 20% intralipid (medium-chain and long-chain fat emulsion injection C6~C24, Pharmaceutical) within 10 min before aortic cross-unclamping, and the control group received an equivalent volume of normal saline. The primary endpoints are complex morbidity of major complications during hospitalization and all-cause mortality within 30 days after surgery. The secondary endpoints include (1) all-cause mortality 6 months and 1 year postoperatively; (2) the quality of life within 1 year after surgery, using the QoR-15 questionnaire; (3) the postoperative cardiac function evaluated by LVEF, LVEDS, and LVEDD, and the myocardial injury evaluated by CK-MB, cTnT, and BNP; and (4) short-term clinical outcomes during hospitalization and total cost are also detailed evaluated.

**Discussion:**

The iCPB trial is the first to explore ILPC on the clinical outcome of adult cardiac surgery patients. The results are expected to provide potential evidences about whether ILPC could reduce the morbidity and mortality and improve the cardiac function and quality of life. Therefore, the results will provide a rationale for the evaluation of the potentially clinically relevant benefit of intralipid therapy.

**Trial registration:**

Chictr.org.cn ChiCTR1900024387. Prospectively registered on 9 July 2019.

## Background

Myocardial ischemia/reperfusion (I/R) injury [1] is an important factor affecting cardiac function and prognosis in patients of cardiac surgery. The underlying mechanisms include oxidative stress, inflammatory reaction, calcium overload, ion channel dysfunction and increased membrane permeability, energy metabolism disorders, etc. [1]. These mechanisms interrelated with each other, and most importantly, the energy metabolism disorders are the initial link. Improving myocardial energy metabolism can reduce myocardial I/R injury [2]. Since the myocardium relies mainly on fatty acid oxidation (FAO) instead of glycometabolism to provide energy, some experimental studies indicated that supplementing the exogenous FAO substrate can increase the supply of myocardial ATP and thus exert cardioprotection [3–7]. However, preischemic exposure of fatty acids will lead to insufficient FAO during the period of ischemia and cause cardiotoxicity injury subsequently. In the meanwhile, clinically, the myocardial ischemic processes are unpredictable, so the intervention of FAO during the period of reperfusion is more controllable and valuable.

Intralipid, a necessary fatty acid carrier, has been safely used as an energy supplier in the clinic for more than 50 years. It has also played an important role in rescuing the cardiac arrest caused by local anesthetic toxicity, which has been incorporated into several clinical guidelines [8–11]. In recent years, experimental studies have shown that lipid emulsion infusion just before reperfusion (i.e., intralipid postconditioning (ILPC)) could reduce myocardial infarct sizes, improve cardiac function, and reduce myocardial I/R injuries [12–16]. Subsequent mechanistic studies define that ILPC acts through the phosphorylation of glycogen synthase kinase-3β via PI3K/Akt/ERK pathways [12, 14, 15] and Caveolin2/STAT3/GSK-3β pathway [13] and ultimately inhibits the opening of mitochondrial permeability transition pore (mPTP), which is essential to mitochondrial calcium overload and reactive oxygen species (ROS). Meanwhile, Umar et al. [16] reported that the cardioprotective effects of lipid emulsion are mediated through G-protein-coupled receptor-40 (GPR40) in two animal models of I/R injury and bupivacaine-induced cardiotoxicity. On the other hand, it could simply be a metabolic switch from glucose to fatty acid metabolism that paradoxically protects the heart [7, 17]. Therefore, despite these interesting experimental findings, the potential clinical usage of intralipid in preventing myocardial I/R injury needs to be further investigated.

Based on the above background, our research group has innovatively conducted a pilot randomized controlled trial (RCT) to investigate whether ILPC would display the same cardioprotective effects in patients of cardiac surgeries, who were inevitably subject to I/R during cardiopulmonary bypass (CPB) [18, 19]. The results showed that ILPC could reduce the release of myocardial damage biomarkers of serum cardiac troponin T (cTnT) and creatine kinase-MB (CK-MB) after cardiac valve replacement surgery, and the total 72-h postoperative area under the curve (AUC) of cTnT and CK-MB were significantly reduced by 32.3% and 26.4% compared with control, respectively [19]. However, due to the relatively small sample content, there were no significant differences between the short-term clinical prognostic parameters such as LVEF, vasoactive inotropic drug score (VIS score), ICU time, length of hospital stays (LOS), morbidity, and mortality within 3 months after surgery.

Therefore, we design a large sample RCT to further study whether intralipid postconditioning could improve the short-term and long-term clinical prognosis as well as cardiac function of adult cardiac surgery patients undergoing cardiopulmonary bypass.

## Objective

Despite the numerous studies on the myocardial protective effects of different drugs and measures, there are only a handful of drugs and measures that can be safely and effectively applied to clinical practice [1, 20]. Our research is a large sample RCT conducted to assess whether intralipid postconditioning undergoing cardiopulmonary bypass surgery will reduce the all-cause mortality within 30 days after surgery and the rate of serious complications during hospitalization and improve the patient’s postoperative cardiac function. The outcome is expected to be the foundation for the future multicenter clinical trial to evaluate the potentially clinically relevant benefit of intralipid therapy. As has been safely and widely used in clinical practice, intralipid may play a novel and important role as a therapeutic intervention for ischemic heart diseases, if similar cardioprotective effects and better clinical outcomes are demonstrated clinically.

## Methods and design

### Study design

**Fig. 1:**
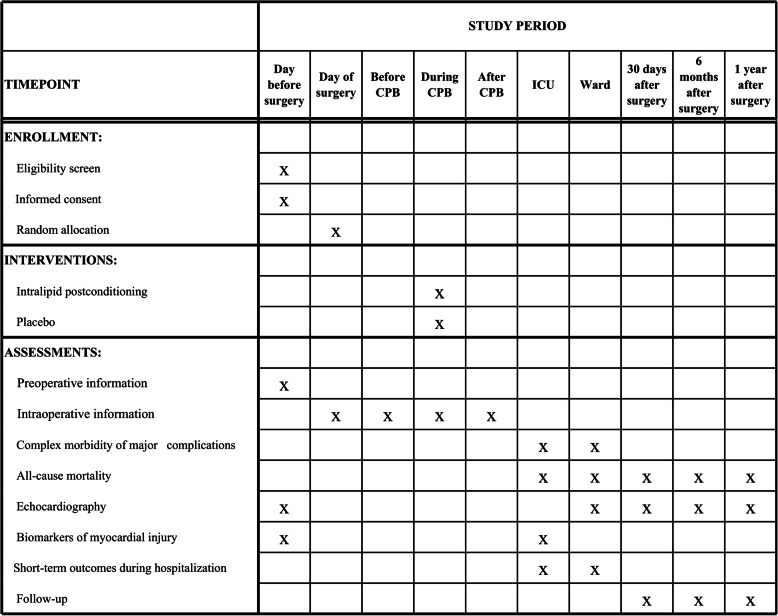
Schedule of enrollment, interventions, and assessments according to Standard Protocol Items: Recommendations for Interventional Trials (SPIRIT)

**Fig. 2:**
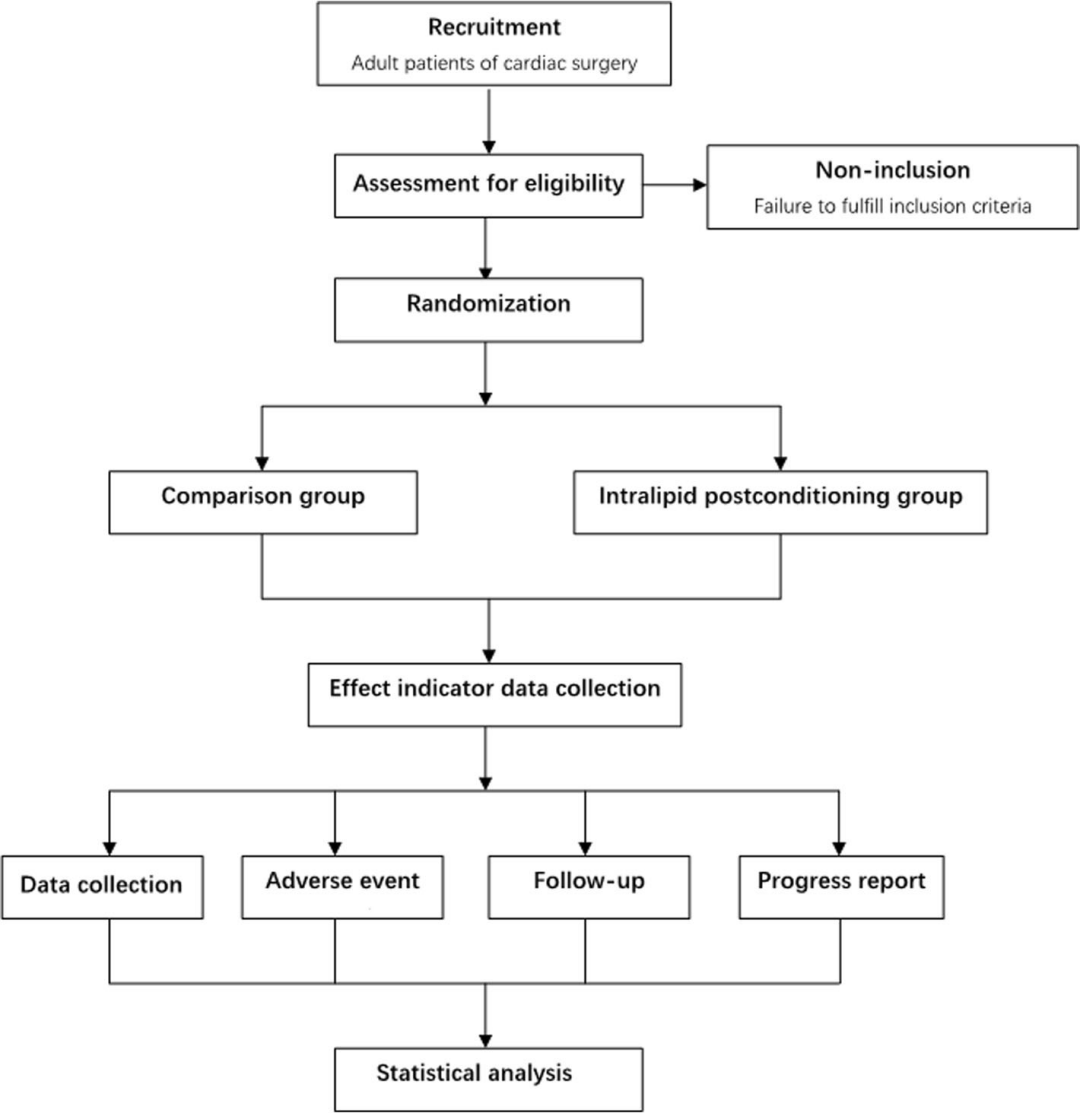
Flow chart

This iCPB study is a large sample, single-center, prospective, double-blinded, two-armed, randomized controlled trial with a 1:1 allocation ratio, testing the clinical outcome of intralipid postconditioning in adult cardiac surgery patients undergoing cardiopulmonary bypass. The protocol structure is written according to the Consolidated Standards of Reporting Trials (CONSORT) 2010 Statement guidelines and follows the Standard Protocol Items: Recommendations for Interventional Trials (SPIRIT) Statement. The SPIRIT checklist can be found in Additional file 1. The SPIRIT figure is illustrated in Fig. 1, and the flow chart in Fig. 2. The study will be conducted in West China Hospital of Sichuan University, China. More than 2500 open-heart procedures with CPB are performed there each year.

### Ethical approval and trial registration

This clinical study protocol has been approved by the Biomedical Research Ethics Committee of West China Hospital of Sichuan University on 4 July 2019 (approval number 2019(324)). The trial has been prospectively registered in the Chinese Clinical Trials Registry (ChiCTR) (www.chictr.org.cn) with the registration number ChiCTR1900024387 on 9 July 2019.

### Participants

We plan to enroll 1000 participants undergoing a cardiopulmonary bypass (CPB) for any elective cardiac surgical procedure via a median sternotomy such as procedures that involve the valves, coronary arteries, or aorta, or combined procedures. Written informed consent will be obtained from all patients before inclusion. The consent form and patient information materials are available from the corresponding author on request.

### Inclusion criteria

Patients will be eligible for enrollment if they meet all the following criteria:

1. Aged 18–70 years old
2. Scheduled to undergo selective cardiac surgery with median sternotomy under CPB
3. Classified by the American Society of Anesthesiologists (ASA) physical status classification scheme of classes I–III
4. Signed informed consent

### Exclusion criteria

Patients who meet any of the following criteria will be excluded from participation:

1. Redo cardiac surgery
2. Experienced cardiogenic shock or cardiac arrest, left ventricular ejection fraction (LVEF) less than 30%, positive baseline serum cTnT or CK-MB
3. Aortic arch and other deep hypothermic circulatory arrest surgery, such as pulmonary thromboendarterectomy
4. Heart transplantation
5. Uncontrolled hypertension (systolic pressure ≥ 180 mmHg or diastolic pressure ≥ 110 mmHg)
6. Hyperlipidemia (TC ≥ 6.2 mmol/L or LDL-C ≥ 4.1 mmol/L)
7. Significant hepatic (international normalized ratio > 2.0), pulmonary (forced expiratory volume-1 < 40% predicted, PaO2/FiO2 < 120) or renal disease (serum creatine level > 150 μmol/L)
8. Severe coagulation dysfunction (Plt, PT, APTT, TT, FIB, and D-dimer were abnormal at the same time.)
9. Any disorder associated with immunological dysfunction (e.g., malignancy or positive serological test for the HIV) in the last 6 months
10. Current infections (meet at least two of the four SIRS diagnostic criteria: body temperature > 38 °C or < 36 °C, HR > 90/min, R > 20/min or PCO2 < 32.33 mmHg, WBC > 12 × 10^9^/L or < 4 × 10^9^/L or immature WBC > 10%)
11. Pregnant women
12. Preoperative treatment with intralipid in the last 1 month
13. Preoperative treatment with nicorandil (an adenosine triphosphate-sensitive potassium channel opener) or sulfonylurea (an adenosine triphosphate-sensitive potassium channel blocker)
14. Participating in other interventional studies

### Randomization/blinding

Patients who meet the enrollment criteria will be randomized 1:1 to either control or ILPC group. Randomization will be performed with the use of a computer-generated randomization sequence from a random number table. The investigator who is responsible for grouping visits the patients the day before surgery and gets the informed consent. Once the patient is qualified, he determines the serial number and the random number of the patient and writes on the case report form (CRF). Any intraoperative event or deviation from the protocol is recorded on the CRF. Allocation concealment was maintained until the time of CPB initiation by using opaque, numbered, and sealed envelopes. Other researchers, such as data collectors and data analysts, will be unaware of the patient grouping. The perfusionists will be aware of patients’ group allocation because they will provide the trial intervention, but they will not be involved in either the postoperative treatment or the analysis. Due to the color difference between intralipid and saline, the intravenous infusion of the two fluids will be shielded.

The patients, surgeons, ICU physicians, data collectors, and data analysts are not aware of the trial grouping in the whole process; if patients are seriously ill, then unblinding will need to be undertaken. Any intraoperative event or deviation from the protocol is recorded on the CRF.

### Interventions

Patients who meet the enrollment criteria will be randomized 1:1 to either the control or the intralipid postconditioning (ILPC) group.

Patients in the ILPC group will receive an intravenous infusion of 2 mL/kg of 20% intralipid (medium-chain and long-chain fat emulsion injection C6~C24, Pharmaceutical) less than 10 min before aortic cross-unclamping. Intralipid should be infused over 10 min in constant speed, and standard or usual care will be given to patients before and after the intervention. The dose of intralipid is chosen on the basis of the bolus dose when it is used in rescuing the cardiac arrest caused by local anesthetic toxicity [8–11]. Patients in the control group received an equivalent volume of normal saline 10 min before aortic cross-unclamping. There will be no special criteria for discontinuing or modifying allocated interventions.

Three investigators (Hong Yu, Yan Zhang, and Rong-Hua Zhou) will explain the treatment intervention in detail and supervise the compliance of intervention throughout the entire procedure (from maintenance of anesthesia to transport to ICU).

### Study endpoints

The primary endpoints are complex morbidity of major complications during hospitalization (all systemic organ complications are graded as I–V in Table 1) and all-cause mortality within 30 days after surgery. Each individual outcome of the composite endpoint will be analyzed separately.

**Table 1:** Grade of complication outcome of the subjects

Grade of complication outcome of the subjects	
I	Healed after temporary treatment
II	Lead to longer hospital stay
III	Life-threatening, but can be basically recovered during hospitalization
IV	Resulting in injuries for 30 days or longer after surgery, the quality of life is significantly reduced
V	Death within 30 days after the surgery (during the study period)

Note: The end of the operation until 24:00 on the day is 0 days after surgery, and the next day after surgery is 00:01–24:00 and so on

The secondary endpoints include (1) all-cause mortality 6 months and 1 year postoperatively; (2) the quality of life within 1 year after surgery, using the 15-item Quality of Recovery Questionnaire (QoR-15) [21, 22]; (3) the postoperative cardiac function evaluated by LVEF, LVEDS, and LVEDD at time points of 1 week, 1 month, 6 months, and 1 year postoperatively, and the myocardial injury evaluated by CK-MB, cTnT, and BNP at time points of 2 h and 24 h postoperatively; and (4) short-term clinical outcomes during hospitalization (postoperative awakening time, mechanical ventilation time, ICU time, length of hospital stay) and total cost are also detailed evaluated.

### Perioperative management and monitoring

Upon arrival in the operating room, standard monitoring includes 5-lead electrocardiogram (ECG) and pulse oximeter. A peripheral venous cannula will be inserted, and patients will be sedated with midazolam intravenously. A radial arterial cannula will be inserted before anesthesia. Intravenous anesthesia is induced with midazolam (0.1–0.2 mg/kg), sufentanil (0.5–1 μg/kg), and rocuronium (0.6 mg/kg). The trachea will be intubated, and mechanical ventilation will start to achieve an end-tidal carbon dioxide tension of 35–45 mmHg, with limited inhaled oxygen concentration (FiO2) 0.4–0.6 and tidal volume (VT) 10 mL/kg during operation. After the induction, anesthesia will be maintained with continuous infusion of propofol 80–100 μg/kg/min or inhalation anesthesia 0.5–2 minimal alveolar concentration (MAC) combined with dexmedetomidine 0.5 μg/kg/h. Midazolam, sufentanil, and rocuronium will be given as needed. Arterial blood pressure, central venous pressure, and nasopharyngeal temperature will be recorded continuously. After systemic heparinization (3 mg/kg, activated clotting time > 480 s), the ascending aorta and superior and inferior lumen (or right atrium) will be cannulated. Standard CPB with a disposable hollow fiber membrane oxygenator (Affinity NT 541; Medtronic, USA) and a roller pump (Stockert-5, Sorin Group, Germany) will be started with a target output of 2.4~2.8 L/min m^2^ of body surface area. After aortic cross-clamping, cardio protection will be provided by cold-blood cardioplegia (1:4) at the dose of 20 mL/kg. The cardioplegia will be repeated half dose every 25 min during surgery. Surgery will be performed under mild hypothermia (33 °C), with MAP 50–70 mmHg and HCT 20–30% during CPB. After the cardiac surgical procedure, the heart will be defibrillated after aortic unclamping, if sinus rhythm does not resume spontaneously. After weaning from CPB, protamine will be used to reverse the effect of heparin. Patients will be transferred to ICU after surgery and extubated at the earliest clinically appropriate time when their ventilation, hemodynamics, and neurological status are considered stable by the attending physician.

### Safety management

The time, management strategies, and consequences of all adverse events will be recorded in the CRFs throughout the entire medical process. Intralipid is widely used in nutritional support for surgical patients and has been used in the clinic for more than 50 years. According to the instructions for the use of its drugs, there may be side effects such as allergic reactions, fever, dyspnea, and other toxic side effects, but the incidence is low. When the adverse reaction of intralipid occurs, the infusion of intralipid should be stopped immediately, patients will receive active, free treatment, and all costs will be covered by our project.

### Postoperative follow-up

Each patient will receive follow-up calls at 1 month, 6 months, and 1 year after surgery to answer questions regarding the presence and quality using the 15-item Quality of Recovery Questionnaire (QoR-15) [21, 22] and to provide the cardiac function data. Each patient will leave at least three phone numbers and receive a maximum of three telephone calls if contact could not be made.

### Data collection

All the related data are collected on the CRF. Preoperative data include general information about age, height, weight, and current and past medical history, especially heart disease history. The classification of the American Society of Anesthesiologists (ASA) was used to assess the risk within 24 h before surgery. Intraoperative data include the type and duration of surgery procedure, duration of CPB and aortic cross-clamp, method of anesthesia, and duration of anesthesia. Postoperative data include the morbidity and mortality; cardiac function evaluated by LVEF, LVEDS, and LVEDD; myocardial injury evaluated by CK-MB, cTnT, and BNP; short-term clinical outcomes during hospital stay (postoperative awakening time, mechanical ventilation time, ICU time, length of hospital stay); and total cost. The follow-up data include quality of life at three time points (1 month, 6 months, and 1 year), using the 15-item Quality of Recovery Questionnaire (QoR-15) and the echocardiography to evaluate the cardiac function.

### Data management and quality control

The original data will be recorded in the CRFs accordingly and maintained confidential. The study supervisor (Rong-Hua Zhou) will supervise the trial conduction every month, and there will not be a data monitoring committee (DMC). The data management and statistical analysis will be performed by the Center for evidence-based medicine and epidemiology, West China Hospital, Sichuan University. Because intralipid is a commonly used medicine in clinical anesthesia, and there have been no reports of serious adverse events, we will not perform an interim analysis.

## Statistical considerations

### Sample size estimate

PASS 15.0 software (NCSS Corp., Kaysville, UT, USA) is used to calculate the sample size. The sample selected for this trial was based on the results of a meta-analysis published by our research team, exploring the influences of inhalation anesthesia compared with total intravenous anesthesia on the patients’ outcomes after valve surgery [23]. The study included 13 randomized controlled trials (a total of 962 patients undergoing heart valve surgery) and found that the 30-day mortality rate was 5.3% and complex morbidity of major postoperative complications was 27%. Taking the complex morbidity of major complications in the meta-analysis as a control, the study has 80% power to detect a 30 relative risk reduction for the primary outcome of complex morbidity at a significance level (alpha) of 0.05 (two-sided), with an allowance of 10% of patients lost to follow-up or withdrawn from the study; finally, the calculated sample size of this study is about 1000 cases (500 cases each group).

### Data analysis

SPSS 22.0 software (IBM Co., Armonk, NY, USA) will be used to analyze the data. Baseline characteristics will be compared using chi-squared or Fisher’s exact tests. The continuous variables that have a normal distribution will be presented as the mean ± standard deviation (SD) or numbers (percentages) and compared using the *t* test or nonparametric test. The incidence of primary and secondary endpoints was measured using the chi-squared test or Fisher’s exact tests. A sensitivity analysis using the modified intention to treat (mITT) approach will also be performed. As a composite outcome, each individual outcome should be analyzed individually. Subgroup classification will be performed according to different types of surgery, Cox analysis will be used to analyze the mortality of different types of surgery, and multivariate logistic regression analysis will be used to correct for risk factors. Variables tested in the model will be selected if *P* < 0.10 or if they are clinically relevant. Results are considered statistically significant if *P* < 0.05. Two-sided significance tests will be used throughout.

## Discussion

Despite advances in surgical technique, anesthesia management, and CPB technology, myocardial I/R injury is still an important factor affecting patients’ cardiac function and clinical prognosis. Ischemic preconditioning and/or postconditioning [24, 25] has been reported to be effective in reducing myocardial I/R injury. This method did not translate into clinical practice partially due to difficulty in the application of ischemic conditioning [25]. Remote ischemic preconditioning (RIPC) prior to cardiac surgery is non-invasive, but recent clinical studies and meta-analysis did not show its benefits on clinical outcomes [26]. Therefore, pharmacological preconditioning or postconditioning has better research and clinical prospects. Even multiple drugs were assessed [1, 20], but none proved to be beneficial in large-scale studies for myocardial protection. Now, intralipid may play a novel and potential role in cardioprotection with bright prospect [3–7].

Intralipid, a safe fat emulsion for human use, is used commonly as a component of parenteral nutrition in clinical practice. It also is used as therapy for severe cardiotoxicity secondary to accidental overdose of local anesthetics, an effect that has been confirmed in animals and humans [8–11]. Because patients with local anesthetic-induced cardiac arrest are considered to be less responsive for standard resuscitation methods, currently, infusion of lipid emulsion is considered the primary treatment for local anesthetic toxicity [10, 11]. In recent years, another striking experimental finding is that intralipid postconditioning (ILPC) could reduce myocardial I/R injuries and thus improve cardiac function, where intralipid was administered as a bolus at the onset of reperfusion [12–17]. So, intralipid may represent a novel and clinically feasible cardioprotective strategy that is highly effective in remodeled hearts. Does the protective effect of lipid emulsion play some role in the effects of propofol (where 10% lipid emulsion is the vehicle) that have been noted in myocardial I/R injury [27]? Therefore, it is necessary that the cardioprotective effects of intralipid need to be clinically verified separately.

Our research group has conducted a pilot study and showed promising results that ILPC induced a significant reduction of postoperative cTnT and CK-MB release in patients undergoing cardiac valve replacement surgery [18, 19]. Moreover, a single intravenous bolus of intralipid (2 mL/kg, 20% intralipid) did not bring abnormal lipid metabolism and was found to be safe, with no perioperative hepatic or renal dysfunction or any other significantly related complications [18, 19]. However, the sample size of the pilot study is small, and it is uncertain to know its influence on patient outcome. Here, we continue to conduct a large sample RCT to further study whether it could improve the cardiac function, the short-term and long-term clinical prognosis of adult cardiac surgery patients, not limited to valve replacement surgery. To our knowledge, this is the first clinical trial investigating the effects of intralipid postconditioning on patient outcome, including mortality, morbidity, and long-term life quality. Our data will provide a rationale for the evaluation of the potentially clinically relevant benefit of intralipid therapy.

Our research has certain limitations. First of all, due to intralipid being white emulsion, it was difficult to achieve the blinding of anesthetists and perfusionists. So even they will provide the trial intervention, but they will not be involved in either the postoperative treatment or the analysis. Secondly, since the inhaled anesthetic sevoflurane is considered to be significantly cardioprotective and the high-dose propofol has also been reported to have a certain myocardial protective effect [27–29], both of which are currently widely used in clinical anesthetics, how to reduce their interference on the results of intralipid postconditioning is of great importance. In our previous pilot study [18, 19], all included patients were given total intravenous anesthesia to eliminate the interference of sevoflurane. However, this does not rule out the role of propofol and is also not consistent with clinical practice. Therefore, in this large sample RCT, we do not limit the anesthesia methods, but will make strict statistics on the dosage of various anesthetics in the intervention group and the control group, so as to ensure the baseline data be comparable. Thus, the confounding factors and interference of the anesthetics will be effectively avoided, and at the same time, it was more consistent with the clinical routine.

In summary, this large sample RCT will be the first to explore the intralipid postconditioning on the clinical outcome of adult cardiac surgery patients. The results are expected to provide potential evidences about whether intralipid postconditioning could reduce the morbidity and mortality and improve the cardiac function and quality of life. Therefore, the results will provide a rationale for the evaluation of the potentially clinically relevant benefit of intralipid therapy.

### Trial status

The trial was started after we obtained the approval of the local ethics committee and registered in the Chinese Clinical Trials Registry. The current protocol is version 2.0 and was issued on 1 June 2019. The study opened to patient recruitment in July 2019. Completion of this trial is expected in December 2021.

## Supplementary information

**Additional file 1.** SPIRIT 2013 Checklist: Recommended items to address in a clinical trial protocol and related documents*.

## Data Availability

During the study, the datasets used in the current study are available from the corresponding author on reasonable request. After the study, the results of this trial will be published in peer-reviewed journals and presented at national and/or international conferences.

## Abbreviations

CPB: Cardiopulmonary bypass
ILPC: Intralipid postconditioning
I/R: Ischemic/reperfusion
RCT: Randomized controlled trial
FAO: Fatty acid oxidation
GPR40: G-protein-coupled receptor-40
cTnT: Cardiac troponin T
CK-MB: Creatine kinase-MB
AUC: Area under the curve
VIS score: Vasoactive inotropic drug score
CONSORT: Consolidated Standards of Reporting Trials
SPIRIT: Standard Protocol Items: Recommendations for Interventional Trials
LOS: Length of hospital stays
LVEF: Left ventricular ejection fraction
LVEDD: Left ventricular end-diastolic dimension
LVEDS: Left ventricular end-systolic diameter
BNP: Brain natriuretic peptide
ECG: Electrocardiogram
VT: Tidal volume
MAP: Mean artery pressure
HCT: Hematocrit
ICU: Intensive care unit
ASA: American Society of Anesthesiologists
CRF: Case report form
PaO2: Arterial blood partial pressure of oxygen
FiO2: Inhaled oxygen concentration
